# Review of a controversial treatment method in the fight against COVID-19 with the example of Algeria

**DOI:** 10.1186/s42269-021-00550-w

**Published:** 2021-05-20

**Authors:** Hani Amir Aouissi, Mostefa Ababsa, Aissam Gaagai

**Affiliations:** 1 Scientific and Technical Research Center On Arid Regions (CRSTRA), 07000 Biskra, Algeria; 2 Department of Biology, Faculty of Sciences, Badji-Mokhtar Annaba University, 23000 Annaba, Algeria

**Keywords:** Hydroxychloroquine, COVID-19, Pandemic crisis, Public health, Algeria

## Abstract

**Background:**

As of April 23, 2021, more than 145 million cases and almost 3.07 million related deaths were noted because of the coronavirus (Covid-19) Pandemic. Considering the low rate vaccination, the alternative that divided opinions for a long time is an old medicine called hydroxychloroquine.

**Main body:**

The aim of this review was to synthesize the different highlights of the most important studies published since the beginning of the epidemic crisis. After a precise study of the available bibliography dealing with this subject and the addition of an adapted example, which is the current situation of Algeria, the results showed the effectiveness of the Algerian method as well as the impact that this treatment had.

**Conclusion:**

We concluded that in brief, given the inexistence of a better solution, we ultimately recommend that patients with severe COVID-19 to be treated for the moment with Hydroxychloroquine combined with Azithromycin in view of its effectiveness, while waiting for another solution.

## Background

Since the beginning of the pandemic crisis (Covid-19), the name of an old drug has come back with insistence, it was hydroxychloroquine (HCQ). This drug was synthesized in the late 1940s (Zhang and Zhong 2020) and the most important point to notice about chloroquine (CQ) and hydroxychloroquine (HCQ) is the fact that have proven effective in the past, particularly in the treatment of malaria.

**Fig. 1:**
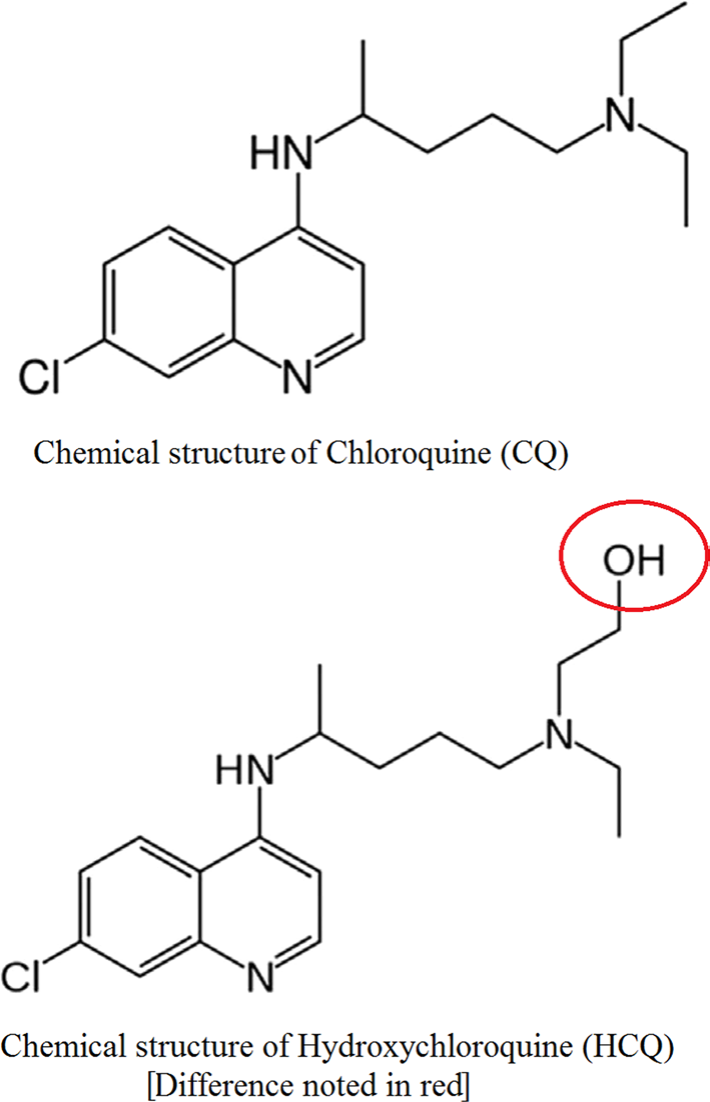
Chemical structures of chloroquine and hydroxychloroquine

CQ and HCQ are synthetic molecules developed from cinchona and are soluble in water (HCQ is more soluble due to the presence of a hydroxyl group –OH) (Faraone et al. 2020) (Fig. 1).

HCQ is primarily a less toxic derivative of CQ known to be effective in inhibiting infection due to SARS-CoV-2 in vitro (Liu et al. 2020). In summary, this is an even safer alternative (Alanagreh et al. 2020). In addition, several studies have suggested that it could prevent endocytosis, among others (Liu et al. 2020; Gautret et al. 2020b; Colson et al. 2020).

According to the CDC (Centers for Disease Control and Prevention), the HCQ was always included in the list of essential drugs (WHO) the “side”' effects had been known for a long time as nausea, diarrhea or occasional vomiting.

## Main text

### Methods and literature review

**Fig. 2:**
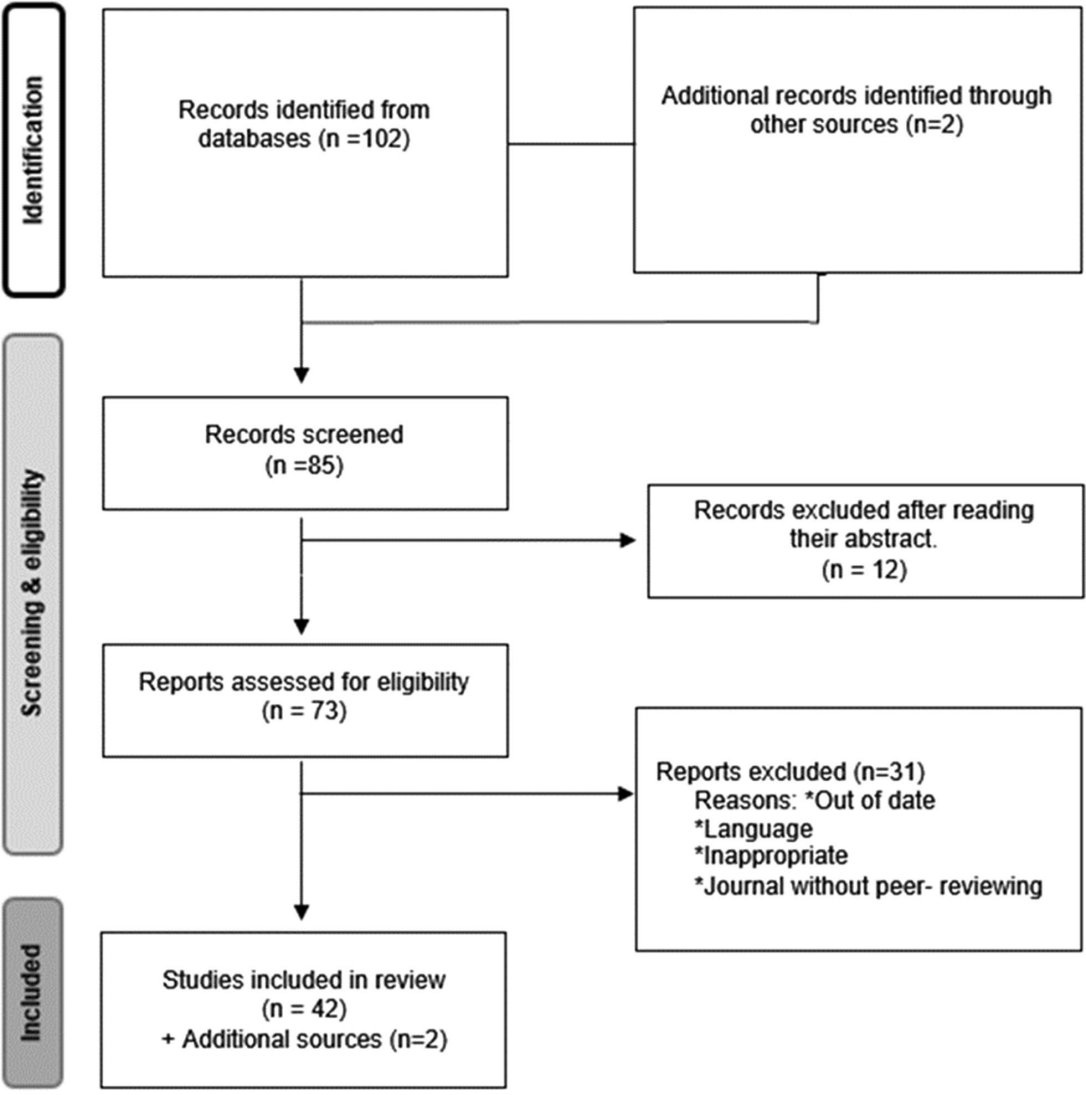
PRISMA flow diagram

For the purpose of this work, we did a research in Internet with the appropriate keywords in the various online databases and websites (Google Scholar, Pubmed, Researchgate, etc.). At the end, a total of 120 papers were selected to be read, between them 85 screened ones, among them 12 were rejected at the abstract level, out of a total of 73 eligible articles, only 42 were selected the other papers were rejected for several reasons (Fig. 2).

This study will come back point by point on the various publications that were for and against this protocol, while giving our opinion by informing on the actual situation of Algeria, which will support our opinion.

As a reminder, the first report of a clinical use has been done in China in February 2020 (Gautret et al. 2020a), which was a draft of some results demonstrating the combined effectiveness of HCQ with Azithromycin in reducing viral infection.

In general, despite having reduced, the exceptional results obtained in particular the first 6 days clearly demonstrated the significant effect of the HCQ-Azithromycin combination (compared to the use of HCQ alone and especially compared to control patients). Going as far as reducing the percentage of PCR positivity to 0 for patients treated with this combination (Gautret et al. 2020). These results are of great importance because an article showed that the average duration of viral shedding in observed patients of COVID-19 in China was 20 days (37 days for the longest duration) (Zhou et al. 2020).

Lecuit (2020) attested that CQ and HCQ have a real antiviral activity on SARS-COV2 in vitro (Lecuit 2020) based essentially on two previous studies (Liu et al. 2020; Wang et al. 2020). At the same time, he has confirmed the successes of Pr. Raoult’s team at that time, warning, however, that these molecules are not devoid of toxicity and that optimal conditions should therefore be applied, specifically to administer medication.

A few days later it was the turn of Boyer et al. (2020) who relied on two other Chinese and French studies, which also affirmed the effectiveness of the protocol (Gautret et al. 2020; Yao et al. 2020) and concluded finally that an analysis of real-life data (hospital information system) could allow to decide even more effectively on the protocol.

At the same time, scientists all over the world have looked into the issue. Among others Wu et al. (2020) in their review have studied the first trials described this protocol as common and stated that several combinations had to be attempted, in particular with dosage variations to optimize the result. Therefore, increasing the number of clinical trials, even if survival rates were already much in favor of using HCQ (Wu et al. 2020).

A few days later, Shukla et al. (2020) shared their opinion after giving a very complete retrospective of the advantages brought by the HCQ. The authors came out with the idea that the number of clinical trials should be increased for even more data if the situation allowed it “ideally.” However, its virulence, the worrying increase in the mortality rate and the spread-growing of the virus, in addition to many other factors. The situation were such that action should be taken instead of allowing as many patients as possible to die needlessly (Shukla et al. 2020). In the meantime, there have been several attempts to combine products and experiments with several different dosages to obtain better results.

The scientific community remains divided. However, the obvious effectiveness of HCQ is unfortunately not unanimous. Many critics were done against the application of this protocol, including (Erickson et al. 2020) criticizing in particular (Gautret et al. 2020). They stipulate that their sample was too small and that the patients were not at the same stage of contamination to provide evidence of any efficacy (something the author himself noted indicating that this allowed better monitoring of the different cases). At the same time, they evoke so-called ethical reasons described by the World Health Organization (WHO) (Erickson et al. 2020), which at that time had stopped clinical trials deeming that more guarantees were needed to keep them going, and then has radically changed its rhetoric many times since the start of the pandemic, banning clinical trials, retracting and banning them again. What would be the (real) reason for this?

The ethical side has been put to the test lately, in particular because of the total containment applied as one of the many preventive measures at a given time of the pandemic, in fact, the state of discomfort, in particular linked to acquired psychotrauma following this confinement, underlined the need for resilience and prevention of consequences (Hervé and Stoeklé 2020).

As Charlier (2020) explained by comparing the Italian model which unfortunately privileged the “economic” aspect of patients before their nature to an individual decision (worst possible choice for the medical profession). And the French model, where each hospital structure has set up a specialized group or an ethics support unit, to make collective decisions (Charlier 2020), which is already much more acceptable from an ethical point of view.

Amiel et al. (2020) have only succeeded in evoking so-called “legal” reasons to demean the study by Gautret et al. (2020) involving, let us keep in mind, clinical trials on 80 people according to which HCQ with Azithromycin were prescribed by the IHU Mediterranean infection team led by Pr. Raoult. However, the authors eventually admitted that in the absence of other alternatives and the urgency of the situation, it was better to act as well as to risk the worst (Amiel et al. 2020).

A considerable number of publications have been published and the same arguments were constantly repeated but this became less and less impactful over time (Savarino et al. 2003)

Considering the situation, it would seem that the main “problem” of HCQ would be the fact that it is very inexpensive and especially already available in almost all countries, its tolerability and its immunomodulatory properties make it logically a solution of choice against viral infections (Erickson et al. 2020). This study has certified its irrefutable effectiveness against viruses in general. An even older study even focused on the fact that chloroquine (Aralen®) or hydroxychloroquine (Plaquenil) were the preferred preventive remedies for travelers to regions affected by malaria (Queyriaux et al. 2008) for quite some time now. Despite everything, it was always specified that it was necessary to use low doses (around 200 mg) 2 times a day (thus reducing the risk of toxicity) instead of high doses cited by the detractors of the protocol (more than 600 mg).

Still according to the same sources, the treatment can be administered to patients of all ages, even pregnant women. Precisely according to Frishman et al. (2019), it turns out that HCQ could be an asset for reducing recurrent early miscarriage (REM). HCQ would have the capacity to lower the risks of pregnancy losses and the associated complications (Frishman et al. 2019) but this possibility has not been exploited so far.

A fact not to be overlooked, the only possible risks would appear in rare cases if the treatment is done in high doses for 5 years on average (Ulviye et al. 2013) (retinopathy, skin diseases, cardiac complications in patients with already progressive cardiovascular pathologies, etc.). Studied in more detail in two other research works (Rynes 1997; Wolfe and Marmor 2010).

The side effects mentioned at the beginning can be greatly reduced, or even, avoided by simply taking the treatment with meals. Objectively, there is therefore no reason to stop the remedy or to ban it, especially after so many years of existence, it is odd, illogical and abnormal that it is only now that we find fault with it. To quote the famous phrase of Dr. Vepachedu from the National Institute of Health in Bethesda (USA): “it’s criminal to say that HCQ is dangerous” (Vepachedu 2020). This same scientist goes further by clearly accusing the WHO of having been grossly mistaken in stopping randomized clinical trials (tests that are logically required to reach an objective conclusion) based essentially on the study by Mehra et al. (2020) (Study retracted later) Was it the pressure? (Media or that of pharmaceutical companies …) or just incompetence? A shocking fact is that the tests resumed later, this did not surprised Pr. Raoult who had effectively noted the appearance on the market of other supposed alternatives (obviously much more expensive) especially the Remdesivir, making our questions legitimate. Thus, while awaiting an effective vaccine, the very interesting prospects of which have been set out in very comprehensive studies (Ghaebi et al. 2020; Amanat and Krammer 2020; Jouneau et al. 2020; Bhattacharya et al. 2020) should serve as a solid basis for the finalization process; several countries are trying to find an antidote or a preventive remedy against this virus. However, at present, all of the treatments tested so far are only aimed at reducing the damage caused by Covid-19, despite the large number of anti-inflammatory and antiviral drugs used. So far, there is no real consensus on treatment and has not been recognized as the ideal solution (Tripathy et al. 2020). A review of all vaccines in phase 3 as well as the perspectives of their applications has been summarized in a very recent study (Funk et al. 2021).

### The case of Algeria

Algeria was among the pioneer countries that adopted the HQC protocol, despite the WHO press releases. After benefiting from the support of the Chinese allies, who have repeatedly said that Algeria was the only country that supported them since the beginning of this crisis when everyone did the opposite. In this sense, the Chinese Ambassador to Algeria, Li Lianhe, highlighted the joint fight led by the two countries against the epidemic while emphasizing the “comprehensive strategic partnership” between the two countries, based on “exceptional friendship and mutual trust” (Dia Algérie).

Algeria was among the countries with the highest death rates in the world, approaching 16% (Ababsa and Aouissi 2020). Then, the trend quickly reversed after using HCQ, going as far as dividing mortality by 3, the precise use of the protocol was described in detail in the reference document by Saadi et al. (2020) to guide health specialists.

Medical care was administered free of charge. All patients were treated the same regardless of their social class or age; in this case, there was no fundamental ethical questioning: everyone, including the most vulnerable, could enjoy the same rights, instead of over-privileging certain categories, according to Piccoli et al. (2020) is the object of a long and hard fight.

Not all this would obviously have been possible without the efforts of the government with a masterly management represented by the President of Algeria Mr. Abdelmadjid Tebboune, and the Minister of Health Pr. Abderrahmane Benbouzid.

At the moment, the borders remain closed while the whole world (including neighboring countries, Morocco and Tunisia) opened too early, these countries are now ravaged and forced to close again (Harizi et al. 2021; Mahrouf et al. 2021).

As of April 2021, the protocol is now only used for severe cases as a last resort (not for mild form). The result is that the protocol allowed 98.2% of the treated patients to recover. Note that no complications have been reported, which proves irrefutably the effectiveness of this protocol when it is correctly applied.

## Conclusions

Ultimately, the aim of this review article was to discuss the most relevant studies by summarizing the information recently published concerning COVID-19 in general and the use of the hydroxychloroquine protocol in a more specific way, as well as different perceptions/interpretations around the world.

It goes without saying that the situation that the world has experienced and still experiencing today can be considered as being unexpected, and therefore, it was difficult to urgently take the right decision with unanimous approval whatsoever, an ethical, legal, clinical point of view, etc. It was, and it will undoubtedly remain, a struggle for health practitioners as well as for the various health and government authorities who have this immense responsibility in their hands. As pointed out (Rhazi and Adarmouche 2020), decisions especially concerning the prescription of drugs must nevertheless be taken in full transparency and communicated to health specialists as well as to the rest of the population.

The main objective of this article was to provide an overview of the most relevant studies by summarizing the information published regarding COVID-19 in general and the use of the hydroxychloroquine protocol more specifically, as well as the different perceptions and interpretations around the world.

Briefly, HCQ has been used for years, it is effective, its side effects are weak and known for a long time, there is no doubt that more tests would ideally be needed to optimize the results, especially in the case of the pandemic to achieve perfect mastery of the protocol. Nevertheless, that sure makes it a potential solution, it will always be better than witnessing a daily massacre. It would seem that the reasons that prevented the world from quickly adopting this protocol, or even applying it, transcend clinical, ethical or even legal reasons. Rather, they are political and/or economic reasons dictated by high institutions whose primary interest is none other than profit.

It would appear that, given the global health crisis we are experiencing now, the option to reallocate QC and HCQ (especially HCQ) in the treatment of SARS-CoV-2 might be a fairly logical approach to follow. The available scientific evidence indicates their diverse mode of action, which irrevocably places them as a solution of choice for the fight against COVID-19, with regard to their pre- and post-infection effects (Ho et al. 2021; Bencedira et al. 2020). However, we must also remain vigilant about the doses administered.

As previously indicated, the molecules are not devoid of toxicity and may incidentally represent a danger to human health if they are poorly administered. However, with the emergence of new evidence, it is necessary to take precautionary measures not to rush to jump to conclusions for the sake of patients with COVID-19. We must wait for the results of prospective, randomized controlled clinical trials determining the ideal doses for a total optimization. While continuing to explore the trail of a vaccine, which let us remember, can only be preventive, a discovery in addition to in-depth and rapid clinical research is necessary. As suggested by Tripathy et al. (2020), therapies based on traditional medicine should even be carefully examined.

At the end, as has been suggested for a long time by Aouissi (2020), in addition to the application of preventive measures that have been repeated at length in recent times. We ultimately recommend that patients with severe COVID-19, especially those with rheumatic diseases (Aouissi and Belhaouchet 2021) to be treated for the moment with hydroxychloroquine combined with azithromycin to cure their infection and limit transmitting the virus to others in order to stop the rapid spread of COVID-19 around the world.

## Data Availability

Data available on request from the authors.

## Abbreviations

COVID-19: Coronavirus disease 2019
CQ: Chloroquine
HCQ: Hydroxychloroquine
CDC: Centers for disease control and prevention
PCR: Polymerase chain reaction
WHO: World Health Organization
REM: Recurrent early miscarriage
